# Draft Genome Sequences of Two Textile Azo Dye-Degrading Shewanella sp. Strains Isolated from a Textile Effluent in Peru

**DOI:** 10.1128/MRA.00836-19

**Published:** 2019-12-05

**Authors:** Ivette Fuentes, Robert Ccorahua, Oscar Tinoco, Oscar León, Pablo Ramírez

**Affiliations:** a Laboratory of Molecular Microbiology and Biotechnology, Faculty of Biological Sciences, Universidad Nacional Mayor de San Marcos, Lima, Peru; b Laboratory of Industrial Effluent Treatment, Faculty of Industrial Engineering, Universidad Nacional Mayor de San Marcos, Lima, Peru; University of Maryland School of Medicine

## Abstract

Here, we report the annotated genome sequences of two Shewanella sp. strains isolated from textile industry wastewater effluent in Peru. Potential genes for encoding enzymes that enable the strain to decolorize and degrade textile azo dyes were detected in both genomes.

## ANNOUNCEMENT

**FIG 1:**
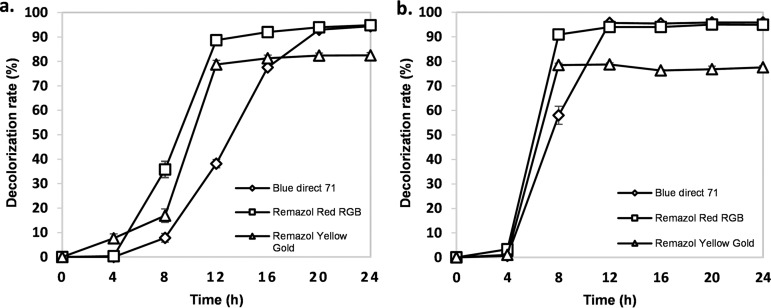
Decolorization of Blue Direct 71, Remazol Red, and Remazol Yellow Gold by *Shewanella* sp. LC2 (a) and *Shewanella* sp. LC6 (b).

Azo dyes are one of the largest classes of synthetic dyes, widely used in the textile industry, and represent about 80% of commercial dyes (1). These dyes are released into the environment through wastewater, due to the high quantities of water used in the dyeing processes (2). Microbial decolorization and degradation of commercial azo dyes are being considered as eco-friendly and cost-competitive alternatives; these use enzymes as azoreductases, laccases, peroxidases, and reductases (3). Shewanella sp. LC2 and *Shewanella* sp. LC6 were isolated from textile industry wastewater effluent in Lima, Peru. Aliquots of 10 ml of wastewater effluent were inoculated into 90 ml of Zhou-Zimmerman liquid medium (4) containing 100 ppm Direct Blue 71, followed by incubation at 30°C under static conditions for 24 h. Colonies were purified by successive streaking onto Tryptic soy (CASO) agar (Merck Millipore) plates and then subjected to decoloration assays. Here, we report the draft genome sequences of strains LC2 and LC6, which exhibited ∼90% to 97% decolorization against Direct Blue 71, Remazol Red, and Remazol Yellow Gold during 24 h of incubation under static culture conditions (Fig. 1).

Bacterial cells from 24-h cultures of strains LC2 and LC6 grown in CASO broth at 30°C were collected by centrifugation (13,000 rpm, 8 min) and used for genomic DNA (gDNA) extraction with a Wizard gDNA purification kit (Promega) following the manufacturer’s instructions. Library construction was performed according to the Illumina TruSeq DNA PCR-free library prep kit protocol with inserts of 550 bp and sequenced using the Illumina HiSeq 2500 platform. The quality of sequencing reads was verified with FastQC (Babraham Institute, Bioinformatics). The assembly was performed with SPAdes v3.11 by using the careful option and increasing k-mer values from 31 to 71 (5). The CAP3 was used to join contigs with identical regions (6). Afterwards, contigs were extended and gap repaired with all paired-end reads by using ABACAS and IMAGE, respectively (7). The quality of assemblies was verified with QUAST (8). Preliminary gene prediction and annotation were performed with the Prokka tool and the NCBI Prokaryotic Genome Annotation Pipeline (PGAP), which uses BLASTp alignments, removing those below thresholds of identity of 25% and coverage of 70% (9, 10). Summary statistics and characteristic features of the draft genome sequencing, assembly, and annotation of the two strains are given in Table 1. The 16S rRNA sequences were compared to the NCBI 16S rRNA database via BLASTN, identifying the best hit to *Shewanella* sp. FDAARGOS_354 for both strains (GenBank accession no. CP022089; query coverage, 100%; identity, 100%).

**TABLE 1:** Characteristics of draft genome sequences and accession numbers of *Shewanella* sp. LC2 and *Shewanella* sp. LC6

	Data for strain:	
Characteristic	LC2	LC6
BioProject no.	PRJNA547647	PRJNA547647
GenBank accession no.	VFSJ00000000	VFSK00000000
SRA no.	SRX6756867	SRX6756866
No. of reads	26,598,558	24,762,742
Read length (bp)	101	101
Genome size (bp)	5,355,693	5,343,011
No. of contigs	147	134
*N*_50_ length (bp)	67,043	69,978
G+C content (%)	46.22	46.21
No. of protein-coding genes	4,941	4,924
No. of tRNA genes	88	93
No. of rRNA genes	21	24

The genomes of both strains presented genes encoding enzymes involved in azo reduction, like a flavin mononucleotide (FMN)-dependent NADH azoreductase and many genes coding for oxidoreductases, thus showing the potential of the strains to perform processes such as decolorization and biodegradation of azo textile dyes. Moreover, the presence of a dye-decolorizing peroxidase (DyP) shows the potential of the strains to degrade not only azo but also high-redox anthraquinone-based dyes (11). Furthermore, genes were found for deamination, nitrate reduction, desulfonation, and sulfate assimilation, as well as for the degradation of benzoate, catechol, protocatechuate, and gentisate, which play a key role in the aerobic degradation of aromatic compounds (12). These characteristics support the capability of *Shewanella* sp. LC2 and *Shewanella* sp. LC6 to biodegrade azo dyes and various xenobiotics.

### Data availability.

The draft genome sequences of *Shewanella* sp. LC2 and *Shewanella* sp. LC6 were deposited in GenBank under the accession numbers listed in Table 1.
